# Ambient Particulate Matter Exposure and Under-Five and Maternal Deaths in Asia

**DOI:** 10.3390/ijerph16203855

**Published:** 2019-10-12

**Authors:** Wei-Hung Lien, Patrick Opiyo Owili, Miriam Adoyo Muga, Tang-Huang Lin

**Affiliations:** 1 Graduate Institute of Space Science and Engineering, National Central University, Taoyuan City 32001, Taiwan; w.h.lien@hotmail.com; 2 Department of Public Health, School of Health Sciences, University of Eastern Africa, Baraton, Eldoret 30100, Kenya; owilip@ueab.ac.ke; 3 Department of Human Nutrition and Dietetics, School of Medicine and Health Sciences, Kabarak University, P.O. Box Private Bag 20157 Kabarak, Kenya; mmuga@kabarak.ac.ke; 4 Center for Space and Remote Sensing Research, National Central University, Taoyuan City 32001, Taiwan

**Keywords:** type of particulate matter, under-five mortality, maternal mortality, Asia

## Abstract

The Asian region is one of the major emission sources of air pollution. Although ambient PM_2.5_ has been linked to several health risks in high-, low-, and middle-income countries, the further analysis of type impact is still rare but significant. The PM_2.5_ distribution retrieved from MODIS (Moderate Resolution Imaging Spectroradiometer) aerosol optical depth products within 16 years thus explored the associations between under-five and maternal mortality for 45 countries in Asia. Both the nonparametric (Generalized Additive Mixed-Effect) and parametric (Generalized Linear Mixed-Effect) models were employed to analyze the collected datasets. The results show that the levels of PM_2.5_ in Asian sub-regions were higher than the Global Air Quality Standards. Biomass PM_2.5_ concentrations was associated with increased the rate of under-five (Incidence Rate Ratio, IRR = 1.29, 95% CI, 1.13–1.47) and maternal (IRR = 1.09, 95% CI: 1.08–1.10) deaths in Asia. Anthropogenic PM_2.5_ was associated with increased rate of under-five deaths in Asia by 12%. The nonparametric method revealed that dust PM_2.5_ was positively associated with the under-five (*β* = 0.04, *p* < 0.001) and maternal (*β* = 0.07, *p* < 0.001) deaths in Asia. The rate of maternal deaths was increased by biomass/dust (IRR = 1.64, 95% CI: 1.63–1.65) and anthropogenic/dust (IRR = 1.22, 95% CI: 1.19–1.26) mixture types. In summary, long-term exposure to different types of ambient PM_2.5_ in high concentration increased the rate of under-five and maternal deaths, suggesting that policies focusing on preventive and control measures is imperative for developing an improved maternal, newborn, and child health in Asia.

## 1. Introduction

Ambient air pollution due to particulate matter (PM_2.5_) in Asia is increasing as a result of rapid growth in population, urbanization, and economies. The World Health Organization (WHO) Global Burden of Disease (GBD) already identified air pollution as a health concern with an estimated 7 million deaths in 2012 resulting from both the ambient and indoor air pollution, of which, South East Asian and Western Pacific regions leading with an estimated deaths of 2.3 and 2.8 million, respectively [1]. PM_2.5_ has also been linked to several health risks as well as increased mortality and morbidity [2,3,4,5,6,7,8]. Hence, concerns about air pollution were reflected in the Sustainable Development Goals (SDGs), with the SDG 11 and the SDG 7 citing air pollution levels in cities and access to clean energy as some of the indicators of the sustainable development energy, respectively, while SDG 3 citing mortality resulting from air pollution as one of the indicators of the health goals [9,10]. 

In 2016, a global assessment of exposure and burden of disease revealed that South-East Asia and Eastern Mediterranean had the highest increase in annual mean PM_2.5_ concentrations from 2008 to 2013 which exceeded the WHO Air Quality Guidelines for annual (10 μg/m^3^) and daily (25 μg/m^3^) mean exposure [9]. However, the effect of this increase in PM_2.5_ on health in the Asia regions has not been adequately explored. Moreover, extant literature also indicates that low-income countries (LMICs) are the most affected regions with several health problems being experienced such as the under-five mortality, which is 11 times higher than that of the high-income countries, and 99% of the global maternal deaths experienced in LMICs [11,12].

A previous study in Africa found that PM_2.5_ from a different source, that is, dust, anthropogenic, and biomass, as well as a combined effect of mixed PM_2.5_, were associated with under-five and maternal deaths [7]. Though plausible, it, however, did not establish the effect of different mixture types of PM_2.5_ concentrations (i.e., dust/anthropogenic mixture, dust/biomass mixture, and anthropogenic/biomass mixture). Our study, therefore, not only explored the relationship between ambient PM_2.5_ and under-five and maternal deaths but also explored the associations between different PM_2.5_ mixture types and the under-five and maternal mortality in Asia.

## 2. Methods

### 2.1. Spatial Domain

**Figure 1:**
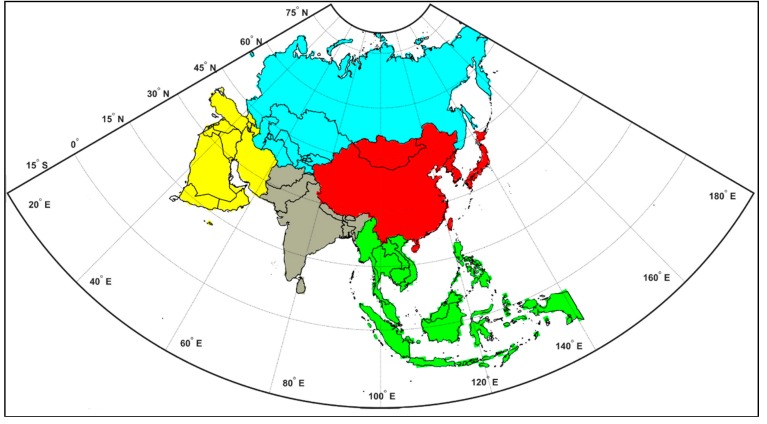
The Mediterranean-Asia which included 45 countries was categorized into five sub-regions in this study as the followings: East Asia (red), Central and North Asia (light blue), West Asia (yellow), South Asia (gray), and Southeast Asia (green).

We included 45 countries in Asia and Mediterranean regions that were geographically categorized into five sub-regions as follows: East Asia (China, Democratic People’s Republic of Korea, Japan, Mongolia, and Republic of Korea), Central and North Asia (Kazakhstan, Kyrgyz Republic, Russian Federation, Tajikistan, Turkmenistan, Uzbekistan), West Asia (Bahrain, Cyprus, Islamic Republic of Iran, Iraq, Israel, Jordan, Kuwait, Lebanon, Oman, Qatar, Republic of Yemen, Saudi Arabia, Syrian Arab Republic, Turkey, and United Arab Emirates), South Asia (Afghanistan, Bangladesh, Bhutan, India, Maldives, Nepal, Pakistan, and Sri Lanka), and Southeast Asia (Brunei Darussalam, Cambodia, Indonesia, Lao People’s Democratic Republic, Malaysia, Myanmar, Philippines, Singapore, Thailand, Timor-Leste, and Vietnam). Figure 1 indicated the five spatial domains of our study with distinct colors for each region.

### 2.2. Under-Five and Maternal Mortality

We used the annual estimated number of under-five and maternal deaths from the year 2000 to 2015. The under-five mortality refers to deaths of children who have not reached the age of five, while maternal mortality refers to deaths of women while they were pregnant or within 42 d of pregnancy termination from any pregnancy-related cause. The number of deaths was limited to residents of specific countries used in this study. However, refugee populations were not included in the country of residence but of their origin. The annual number of all-cause mortality data, which are generally based on the data generated by national statistical systems, from the World Bank were used [13].

### 2.3. Ambient PM_2.5_ Concentration

The Moderate Resolution Imaging Spectroradiometer (MODIS) monthly aerosol optical depth products (MOD08/Terra and MYD08/Aqua) was employed to obtain the annual mean ambient PM_2.5_ concentrations for 16 years (i.e., 2000–2015) in the spatial domain [7,14,15]. These data were collected by the National Aeronautics and Space Administration (NASA). Ambient PM_2.5_ types were derived using spectral derivatives (first and second order, see also Figure S1) of aerosol optical depth (AOD) which differentiated between aerosol types including biomass burning (BB), anthropogenic pollutant (AP), mineral dust (DS), biomass/dust mixture (BD), anthropogenic/dust mixture (AD), and biomass/anthropogenic mixture (BA) as applied in other studies [7,16]. The emission sources of AP, BB and DS aerosols frequently occur in the regions of industrial parks and megalopolis in Asia, Southeast Asia during springtime and summertime, and Middle East Asia during the wet season (April–September), respectively. Both the temporal and spatial exposures were obtained.

### 2.4. Covariates

The indicators used in our study were ranging from environmental factors (i.e., annual mean temperature) to health (i.e., anemia prevalence among pregnant women, undernourished population, TB deaths, and AIDS deaths) and other demographic indicators (i.e., population employed, female population, population living in urban areas, country’s total population, year of study, and country). The temperature was expressed in degrees Celsius of the aggregated country and basin levels to get the annual mean. The relationship between temperature, PM_2.5_ and mortality have been established [17,18,19,20]. The health indicators such as pregnant women with anemia included women diagnosed with hemoglobin level less than 110 grams per liter (g/L) at sea level. The undernourished population was the number of people who continuously had insufficient food intake to maintain dietary energy levels. The TB death rate estimated among the HIV-negative populace was determined per 100,000 populations while AIDS deaths were deaths resulting from AIDS-related causes. All the health-related indicators were included in this study since they can also contribute to the death of both the mothers and their children. Other demographic indicators such as population employed were expressed as a proportion of the 15 years old and above who were employed to indicate the socioeconomic status of the population. The population of females included the number of all female residents based on the national statistics, while the urban population and total population were the number of people living in urban areas and all residents, respectively. The most recent data were considered in our study (i.e., from 2000 to 2015).

### 2.5. Statistical Analysis

The Expectation Maximization (EM) algorithm, which can handle up to 50% of missing data, was employed to impute missing data. The total observation was 720 (i.e., 45 countries by 16 years period) while some of the indicators had missing data and were as follows: temperature (138), pregnant women with anemia (180), population undernourished (272), TB deaths (47), AIDS deaths (230), population employed (45), female population (45), population in urban (45), total population (45). The range of the missing data was between 0.06% and 37.8% for the indicators used in our study. One of the benefits of using the EM algorithm to impute missing data is that it can handle up to 50% of missing data.

Descriptive statistics were presented in terms of the annual means. In the inferential statistics, however, we used both the Generalized Linear Mixed-Effect Model (GLMM) and the Generalized Additive Mixed-Effect Model (GAMM) to explore the associations between PM_2.5_ and the under-five and the maternal mortality. The penalized quasi-likelihood estimation approach was used in the GLMM while a smoothing function, Poisson link function, and a spatial covariance structure were employed in both the GLMM and the GAMM approaches. Since there were variations between different countries and across different years, the country and the year were used in the random-effect models. Two models were analyzed in this study. The first model was unadjusted while the second model was adjusted for all the covariates. Stratified analysis was also employed to determine the associations according to different geographical regions. Incidence rate ratios (IRRs), which is the number of events occurring at any given point-in-time, were obtained and reported. The dose-response relationship between PM_2.5_ and mortality was assessed using the GAMM penalized spline and generalized cross-validation approach to estimate the degree of freedom. We used R version 3.2.2 (R Foundation for Statistical Computing, Vienna, Austria) for our analyses.

## 3. Results

**Figure 2:**
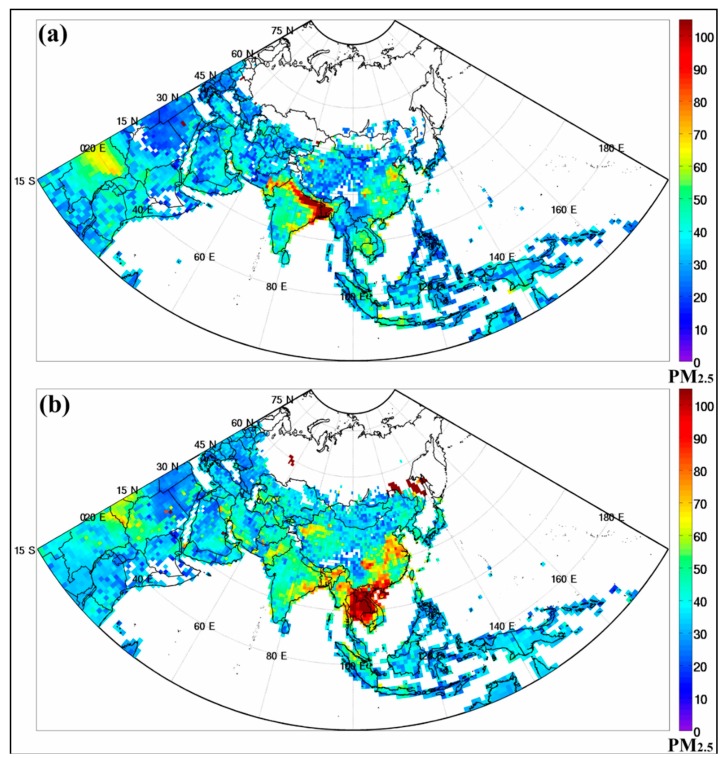
The transition of heavy PM_2.5_ (μg/m^3^) loading from (**a**) South Asia in January 2016 to (**b**) Southeast Asia in March 2016, highly potential due to human activities in different seasons.

According to the observation of MODIS in monthly aerosol products, it was also found that the PM_2.5_ concentrations were high across different time periods in different sub-regions of Asia as shown in Figure 2. Figure 2a shows the PM_2.5_ distribution from anthropogenic pollution in South Asia to be high in January 2016, but after two months, the PM_2.5_ distribution from biomass burning was high in Southeast Asia in March 2016 as shown in Figure 2b. 

Table 1 presents the descriptive results by different Asian sub-regions for the indicators used in our study. All the indicators in the sub-regions were statistically different at *p* < 0.05, except for the indicators female population (*p* = 0.473) AIDS death (*p* = 0.788). The means of the under-five and maternal deaths were high in South Asia at 330,969.9 (*SD* = 605,291.9) and 12,617.7 (*SD* = 23,035.4), respectively. The PM_2.5_ concentrations from biomass burning and dust were high in Southeast Asia at 42.71 μg/m^3^ and 37.88 μg/m^3^, respectively, while the anthropogenic was highest in South Asia (33.52 μg/m^3^). All the mixture type PM_2.5_ was above 40 μg/m^3^ in all the sub-regions.

Table 2 is the unadjusted model of the association between PM_2.5_ and under-five and maternal mortality. The PM_2.5_ types were associated with under-five and maternal deaths, with the under-five children in Asia being at highest incidence of death as a result of biomass and dust mixture PM_2.5_ (IRR = 8.43, 95% CI: 7.98–8.91) while dust PM_2.5_ had the highest incidence rate of maternal deaths (IRR = 3.55, 95% CI: 3.54–3.56). The unadjusted analyses revealed that the incidence of under-five and maternal deaths in all the sub-regions were not statistically different from East Asia sub-region.

In overall, the adjusted GLMM analyses in Table 3 revealed that the annual biomass PM_2.5_ concentrations were associated with the incidence of maternal mortality (IRR = 1.09, 95% CI: 1.08–1.10) and under-five mortality (IRR = 1.29, 95% CI: 1.13–1.47) in Asia, while the annual PM_2.5_ concentrations of biomass/dust mixture (IRR = 1.64, 95% CI: 1.63–1.65) and anthropogenic/dust mixture (IRR = 1.22, 95% CI: 1.19–1.26) types were associated with the incidence of maternal deaths at *p* ≤ 0.05. The nonparametric method (GAMM) however revealed an association in all the PM_2.5_ types with anthropogenic, dust, biomass/dust mixture type and biomass/anthropogenic mixture type being positively associated with the incidence of under-five deaths, while dust and biomass/dust mixture type being positively associated with the incidence of maternal deaths.

**Figure 3:**
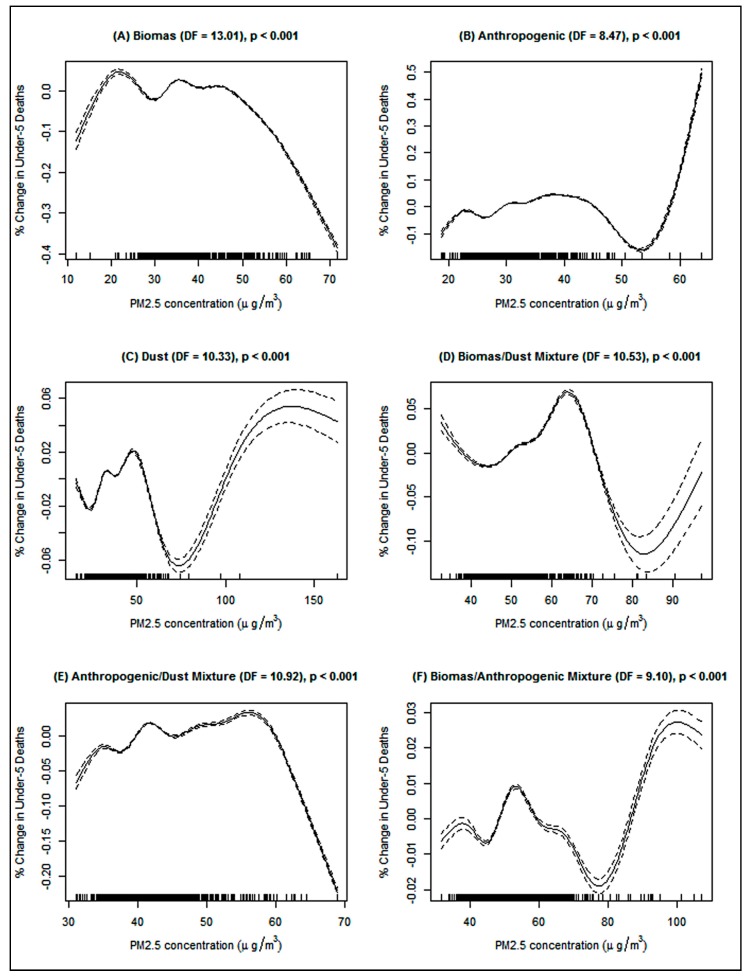
The penalized spline of the association between ambient PM_2.5_ concentration and under-five deaths in Asia. (**A**) Biomass PM_2.5_, (**B**) Anthropogenic PM_2.5_, (**C**) Dust PM_2.5_, (**D**) Biomass/dust mixture PM_2.5_, (**E**) Anthropogenic/dust mixture PM_2.5_, and (**F**) Biomass/anthropogenic mixture PM_2.5_. Adjusted for temperature, undernourished population, TB deaths, AIDS deaths, employed population, female population, urban population, total population, year, and country. The dose-response relationship is represented by the solid line while the broken line represents the 95% confidence interval.

**Figure 4:**
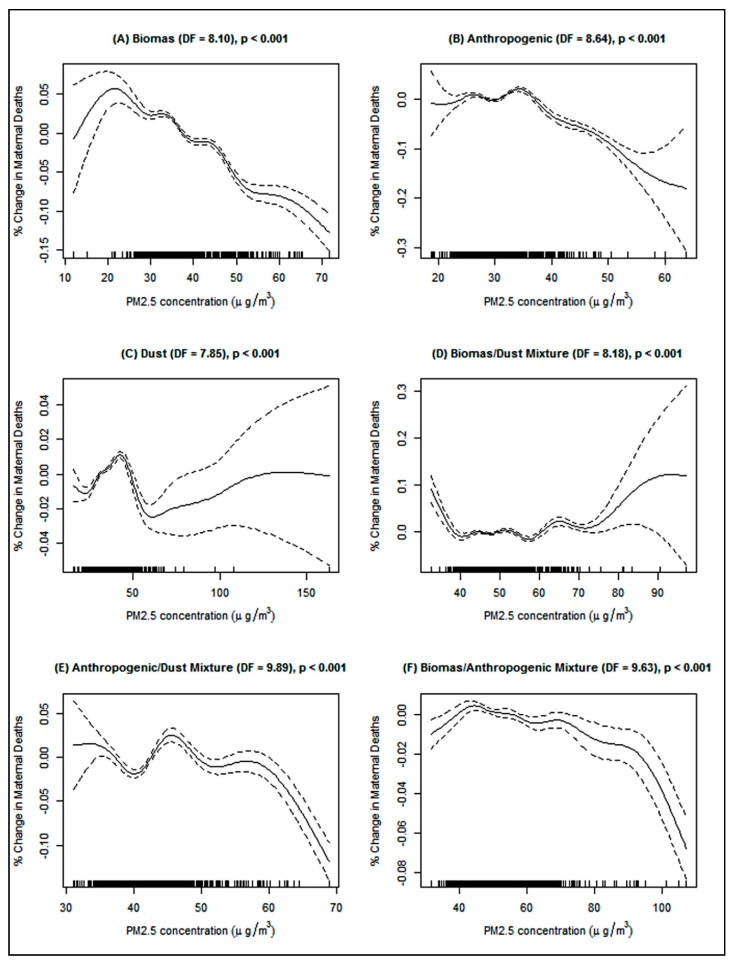
Penalized spline of the association between ambient PM_2.5_ concentration and maternal deaths in Asia. (**A**) Biomass PM_2.5_, (**B**) Anthropogenic PM_2.5_, (**C**) Dust PM_2.5_, (**D**) Biomass/dust mixture PM_2.5_, (**E**) Anthropogenic/dust mixture PM_2.5_, and (**F**) Biomass/anthropogenic mixture PM_2.5_. Adjusted for temperature, undernourished population, TB deaths, AIDS deaths, employed population, female population, urban population, total population, year, and country. The dose-response relationship is represented by the solid line while the broken line represents the 95% confidence interval.

The stratified analyses of the sub-regions revealed that the under-five children in East Asia region only were at 2% increased incidence of death as a result of biomass PM_2.5_ while mothers in Central/North Asia region only were at 7% higher rate of death from biomass PM_2.5_ in both the GLMM and GAMM models (Table 3). For anthropogenic PM_2.5_, and increase death rate of under-five deaths was realized in East Asia (GAMM, *β* = 0.03, *p* ≤ 0.001), West Asia (GLMM, IRR = 1.02, 95% CI: 1.01–1.03) and Southeast Asia (GAMM, *β* = 0.09, *p* ≤ 0.001). Anthropogenic PM_2.5_ was however not associated with an increased death rate among mothers. Using the GAMM model, dust PM_2.5_ was associated with an increased under-five death (*β* = 0.11, *p* ≤ 0.001) and maternal death (*β* = 0.07, *p* ≤ 0.05) in Central/North Asia, and an increased rate of under-five deaths in Southeast Asia (*β* = 0.03, *p* ≤ 0.001). The PM_2.5_ concentrations of biomass/dust mixture type was associated with an increased rate of under-five deaths in Central/North Asia (GAMM, *β* = 0.03, *p* ≤ 0.001), South Asia (GAMM, *β* = 0.04, *p* ≤ 0.001) and Southeast Asia (GAMM, *β* = 0.11, *p* ≤ 0.001). The PM_2.5_ concentrations of anthropogenic/dust mixture type were associated with an increased under-five death rate in West Asia (GAMM, *β* = 0.03, *p* ≤ 0.01) and Southeast Asia (GAMM, *β* = 0.16, *p* ≤ 0.001), and 8% increase incidence of maternal deaths in Central/North Asia only. There was a clear increased incidence of under-five deaths in Southeast Asia as a result of biomass/anthropogenic mixture PM_2.5_ (GAMM, *β* = 0.07, *p* ≤ 0.001) and a marginal increase in under-five deaths (GLMM, IRR = 1.01, 95% CI: 1.01–1.02) in East Asia and West Asia. However, the penalized-spline showed somewhat dose-response relationships between PM_2.5_ types and the under-five mortality (Figure 3) and maternal mortality (Figure 4) which appeared to have different shapes like *n*, *w,* and *v* shaped. Yet, all the splines exhibited some form of non-linear associations. For example, after a biomass PM_2.5_ concentrations of about 19 µg/m^3^ there was an increase in the percentage change in the under-five deaths (Figure 3A) and later a slight increase in deaths after about 32 µg/m^3^. 

## 4. Discussion

Ambient and indoor PM_2.5_ is increasingly becoming one of the major focuses of health-related determinants in Asia and globally [21,22], and our study is one of such that focuses on its health effect in a region that is well known for higher levels of short-term and long-term ambient PM_2.5_ exposure [9]. This study explored the relationship between types of ambient PM_2.5_ and under-five and maternal deaths and found positive associations with under-five and maternal mortality which was consistent with previous studies [5,7,23,24]. The levels of exposure to ambient PM_2.5_ in the Asian sub-regions also exceeded the recommended Air Quality Guidelines of daily (25 μg/m^3^) and annual (10 μg/m^3^) exposure [9,25].

Several sources of air pollution contribute to the high levels of exposure to hazardous chemicals in the air in Asia as well as globally. Dust storms which are hazardous weather events occur frequently in Asia and North Africa, particularly during boreal spring and summer. In addition, Asia and Africa remain two major emission regions of soot aggregate (black carbon dominated), generally associated with automobile, industrial and open combustion (i.e., forest fires, land-clearing by fire, agricultural waste-burning events, and from traditional stoves and kilns). Several studies have also concluded that 50 to 90% of the South Asian aerosol originates from fossil fuel combustion [26,27,28]. We found that the PM_2.5_ concentrations from different sources were high and varied across different time periods in different sub-regions of Asia. For example, we found that PM_2.5_ distributions from anthropogenic pollutants were the highest in South Asia in January. However, after two months, the PM_2.5_ distributions of biomass burning were high in Southeast Asia in March mainly because of agricultural cleanup activities in the springtime, and hence an increase in PM_2.5_ pollution from biomass burning [29,30,31,32]. In general, the change in PM_2.5_ distributions was mainly influenced by seasonal changes in human activity.

In this study, we found that biomass PM_2.5_ in Asia increased under-five deaths (IRR = 1.29) and maternal deaths (IRR = 1.09) significantly. However, stratified analyses revealed that biomass PM_2.5_ mostly affected East Asia (i.e., in under-five deaths) and Central/North Asia (i.e., in maternal deaths). Our findings are not different from a previous study which found that PM_2.5_ concentrations from biomass burning are associated with maternal and under-five deaths in Africa [7]. The point estimates of our stratified analyses may vary from the overall analyses, that is—lower than the overall Asian region, because of a reduced sample size after stratification which may also lead to underestimation of the true effect. Nevertheless, we still found a significant association between chronic exposure to PM_2.5_ and all-cause mortality as evidenced in previous studies [7,33].

Several previous studies that were conducted in different populations found different health effects of PM_2.5_ [4,5,6], but few had focused on the under-five children and their mothers. This may be because of an increased focus on other important mortality-contributing factors which are, generally, clinical in nature such as abortion complications, hemorrhage, infection/sepsis, and hypertensive disorders. Focusing on clinically related deaths is plausible, but this may also lead to neglect of other important factors that may contribute to the under-five and maternal deaths such as the environmental factors and other social determinants. Nonetheless, in this study we found that the under-five children were at 12% increased rate of death from chronic exposure to anthropogenic PM_2.5_ in Asia, and this positive association was only found in West Asia (IRR = 1.02) and Southeast Asia (*β* = 0.09) in the parametric and the nonparametric models, respectively. The anthropogenic pollutants from combustion processes have been however linked to several other health issues such cardiac disease and diseases of the respiratory system and reproductive health in both animals and humans [34,35].

On the other hand, the nonparametric model revealed also a positive association between dust PM_2.5_ and under-five and maternal mortality in Asia by 4% and 7% increased death rate, respectively. The effect of dust particulate matter was higher in Central/North Asia which had 11% and 7% increased death rate among under-five children and mothers, respectively. The PM_2.5_ concentrations from dust were also positively associated with the under-five deaths in Southeast Asia. 

In the PM_2.5_ mixture types, biomass/dust mixture was positively associated with the under-five deaths in Asia, but mainly in the sub-regions of Central/North Asia, South Asia and Southeast Asia in the stratified analyses. For maternal deaths, the point estimate of biomass/dust mixture in the entire Asian region was high at 64% incidence of maternal death, and yet, the stratified analyses did not reveal the most affected sub-region. On the other hand, the incidence rate of maternal deaths from anthropogenic/dust mixture PM_2.5_ was 22% in Asia with a positive association found in the sub-region of Central/North Asia only. There was also a positive association between anthropogenic/dust mixture PM_2.5_ and under-five deaths in West Asia and Southeast Asia sub-regions. With regards to biomass/anthropogenic mixture PM_2.5_, there was a positive association with the under-five mortality in the entire Asian region with a clear positive relationship found in the Southeast Asia sub-region. The positive association between all mixture type PM_2.5_ concentrations and the under-five mortality was found in the Southeast Asia sub-region. This finding is important because mothers and children in Asia may have been exposed to multiple pollutants rather than one type of pollutant from specific source. We also found that there was a higher rate of maternal deaths in Asia with exposure to biomass/dust and anthropogenic/dust mixture types. Some of the extant literature suggests that the biological mechanisms between PM_2.5_ and mortality include reduced lung function, increased blood pressure, and others such as reproductive health [36,37,38,39]. Nevertheless, our remains the first to explore the combined effect of several mixture types of PM_2.5_ on mortality.

### Limitations and Strengths

One major limitation of our study included the use of all-cause mortality because of the inability to determine the cause-specific deaths. These deaths might have been a result of several other related factors that are clinical in nature and may not have been related to environmental factors. Secondly, we could not control for another important confounding environmental and individual factors, such as precipitation, humidity, and demographics of individuals, because of data inadequacy and the ecological nature of our study. Future studies should focus on adequate exposure assessment and individual follow-up to be able to extend the findings of this study. Third, the point estimate may have not reflected the true causality because of the small sample size (i.e., 45 countries with a follow-up period of 16 years), and hence there was lack of statistical power. This may lead to underestimation of the true effect. Therefore, our results should be cautiously interpreted because it only reflects a temporal causality. However, this study is important since it is the first of its kind in Asia to explore the association between different types of PM_2.5_ and the under-five and maternal deaths. Secondly, one of the major challenges for researchers is the estimation of disease-response to ambient PM_2.5_ exposure due to nonlinearity [8]. Our study used both the linear and nonlinear approaches to explore the associations. Finally, our spatial domain was wide enough to understand the Asian region, in light of PM_2.5_ and its general health effect.

## 5. Conclusions

In conclusion, we examined the relationship between PM_2.5_ and under-five and maternal deaths in Asia and found a positive relationship. The level of PM_2.5_ exposures was also high in Asian sub-regions than global standards. Our point estimates may have been underestimated due to spatial variability in PM_2.5_ and the few cases in our data. Data from some Asian regions are not easily available. Hence, our study adds more knowledge to the current body of literature by highlighting the need for more research to enhance adequate policies development focusing on preventive and control measures of PM_2.5_ exposures.

## Figures and Tables

**Table 1:** Descriptive statistics of long-term under-five and maternal deaths, aerosol optical depth (AOD), types of PM_2.5_ and population characteristics, by sub-regions of Asia.

		East Asia	Central/North Asia	West Asia	South Asia	Southeast Asia	*p*-Value ^a^
		Annual Mean (*SD*)	Annual Mean (*SD*)	Annual Mean (*SD*)	Annual Mean (*SD*)	Annual Mean (*SD*)	
**Under-5 deaths**, *n*		73,791.6 (149,993.4)	14,375.2 (9,801.0)	11,758.1 (16,626.0)	330,969.9 (605,291.9)	38,592.6 (52,828.0)	<0.001
**Maternal deaths**, *n*		1445.5 (2708.6)	193.7 (185.1)	357.7 (812.1)	12,617.7 (23,035.4)	1,622.32 (2672.2)	<0.001
**Biomass**							
	AOD, *τ*	0.29 (0.07)	0.25 (0.06)	0.30 (0.11)	0.30 (0.08)	0.35 (0.10)	<0.001
	PM_2.5; Jan-Dec_, μg/m^3^	37.84 (6.12)	32.76 (3.54)	35.51 (7.99)	38.54 (6.32)	42.71 (8.08)	<0.001
**Anthropogenic**							
	AOD, *τ*	0.37 (0.10)	0.30 (0.06)	0.32 (0.12)	0.40 (0.11)	0.35 (0.07)	0.024
	PM_2.5; Jan-Dec_, μg/m^3^	32.19 (5.40)	28.24 (2.99)	29.53 (6.20)	33.52 (5.92)	31.10 (3.91)	0.009
**Dust**							
	AOD, *τ*	0.48 (0.35)	0.45 (0.26)	0.40 (0.11)	0.48 (0.15)	0.51 (0.17)	0.008
	PM_2.5; Jan-Dec_, μg/m^3^	36.94 (17.54)	35.50 (12.53)	32.26 (5.72)	36.50 (7.68)	37.88 (8.81)	0.024
**Biomass/Dust Mixture**							
	AOD, *τ*	0.28 (0.06)	0.33 (0.08)	0.37 (0.14)	0.38 (0.12)	0.32 (0.08)	0.040
	PM_2.5; Jan-Dec_, μg/m^3^	45.15 (4.26)	47.14 (4.50)	49.97 (9.15)	51.64 (7.39)	48.71 (5.58)	<0.001
**Anthropogenic/Dust Mixture**							
	AOD, *τ*	0.39 (0.09)	0.35 (0.07)	0.36 (0.10)	0.48 (0.14)	0.37 (0.09)	0.006
	PM_2.5; Jan-Dec_, μg/m^3^	42.67 (5.02)	40.52 (3.56)	40.92 (5.03)	47.76 (7.39)	41.74 (4.60)	0.004
**Biomass/Anthropogenic Mixture**							
	AOD, *τ*	0.41 (0.15)	0.28 (0.08)	0.36 (0.16)	0.42 (0.14)	0.48 (0.21)	<0.001
	PM_2.5; Jan-Dec_, μg/m^3^	53.74 (9.64)	45.34 (5.26)	49.56 (9.25)	53.37 (8.10)	57.76 (13.24)	<0.001
**Total population**, million		291.0 (498.0)	37.6 (50.4)	22.6 (28.4)	188.0 (368.0)	54.7 (64.3)	<0.001
**Urban population**, million		146.0 (224.0)	22.9 (37.0)	13.9 (16.6)	57.2 (111.0)	23.4 (30.5)	<0.001
**Female population**, %		50.15 (1.07)	51.01 (1.38)	44.96 (7.15)	49.05 (1.33)	49.94 (0.98)	0.473
**Employed population**, %		63.59 (7.54)	58.42 (4.14)	52.36 (14.01)	59.31 (11.52)	65.92 (10.47)	<0.001
**AIDS death**, thousand		12.3 (10.2)	3.2 (3.9)	4.1 (2.8)	3.3 (4.8)	9.3 (10.8)	0.788
**Tuberculosis death rate**, per 100,000		16.27 (26.86)	15.91 (6.76)	2.91 (5.07)	32.23 (23.03)	40.42 (36.64)	<0.001
**Undernourished population**, million		42.0 (73.20)	4.9 (5.0)	6.0 (3.3)	35.0 (64.3)	10.4 (10.0)	0.049
**Anemia in pregnancy**, %		27.43 (3.96)	31.66 (4.36)	31.41 (5.14)	43.89 (8.42)	34.74 (7.98)	<0.001
**Annual mean temperature**, °C		9.46 (5.45)	8.06 (7.25)	22.09 (4.82)	20.30 (5.77)	24.21 (3.27)	<0.001

^a^, Generalized Linear Mixed-Effect was used to test for trend with the year being the random effect, *SD*, Standard deviation.

**Table 2:** Unadjusted generalized linear mixed-effect models of under-5 and maternal mortality in Asia.

Variable		Unadjusted GLMM, IRR (95% CI) ^b^	
		Under-5 Deaths	Maternal Deaths
**Type of PM_2.5_^a^**			
	Biomass	1.45 (1.44, 1.45) ***	1.24 (1.23, 1.24) ***
	Anthropogenic	0.84 (0.77, 0.93) ***	2.65 (2.65, 2.65) ***
	Dust	2.58 (2.57, 2.59) ***	3.55 (3.54, 3.56) ***
	Biomass/Dust	8.43 (7.98, 8.91) ***	3.16 (3.15, 3.16) ***
	Anthropogenic/Dust	0.92 (0.83, 1.01)	1.07 (1.02, 1.12) **
	Biomass/Anthropogenic	2.29 (2.27, 2.30) ***	2.04 (2.03, 2.05) ***
**Sub-regions (ref: East Asia)**			
	Central/North Asia	1.12 (0.11, 11.62)	0.58 (0.05, 6.27)
	West Asia	3.03 (0.04, 2.29)	0.29 (0.04, 2.27)
	South Asia	3.58 (0.39, 32.84)	6.68 (0.71, 62.55)
	Southeast Asia	1.15 (0.14, 9.37)	1.86 (0.22, 15.48)
**Year**		0.44 (0.43, 0.44) ***	0.39 (0.39, 0.39) ***
Total population		0.001 (0.001, 0.001) ***	0.21 (0.01, 0.78)
Urban population		1.20 (1.12, 1.29) ***	1.27 (1.19, 1.36) ***
Female population		2.20 (2.08, 2.34) ***	2.06 (2.02, 2.10) ***
Employed population		0.86 (0.85, 0.86) ***	0.59 (0.59, 0.59) ***
AIDS death		0.08 (0.07, 0.08) ***	0.07 (0.07, 0.07) ***
Tuberculosis death		7.59 (7.54, 7.65) ***	11.82 (11.79, 11.85) ***
Undernourished population		1.17 (1.11, 1.22) ***	1.20 (1.16, 1.24) ***
Anemia in pregnancy		13.92 (6.11, 31.72) ***	30.76 (12.24, 77.29) ***
Annual mean temperature		2.55 (2.53, 2.57) ***	4.56 (4.54, 4.59) ***

^a^, In one unit increment of PM_2.5_ concentration. ^b^, Natural cubit spline smoothing was used. GLMM, Generalized linear mixed-effect model. IRR, Incidence rate ratio. CI, Confidence interval. * *p* ≤ 0.05, ** *p* ≤ 0.01, *** *p* ≤ 0.001.

**Table 3:** Adjusted generalized linear mixed-effect models and generalized additive mixed-effect models of under-5 and maternal mortality by sub-regions of Asia.

		Under-5 Deaths, Adjusted Model		Maternal Deaths, Adjusted Model	
Annual Mean PM_2.5_^a^		GLMM + NS IRR (95% CI) ^b,c^	GAMM + NS Coef. (*Se*) ^b^	GLMM + NS IRR (95% CI) ^b,c^	GAMM + NS Coef. (*Se*) ^b^
**Asia Overall**					
	Biomass	1.29 (1.13, 1.47) ****	−0.20 (0.003) ****	1.09 (1.08, 1.10) ****	−0.25 (0.01) ****
	Anthropogenic	1.12 (1.01, 1.24) **	0.03 (0.004) ****	1.02 (0.97, 1.08)	−0.18 (0.001) ****
	Dust	0.89 (0.77, 1.04)	0.04 (0.003) ****	1.00 (0.99, 1.00)	0.07 (0.01) ****
	Biomass/Dust	0.95 (0.85, 1.05)	0.07 (0.003) ****	1.64 (1.63, 1.65) ****	0.03 (0.01) ****
	Anthropogenic/Dust	1.08 (0.95, 1.23)	−0.04 (0.003) ****	1.22 (1.19, 1.26) ****	−0.08 (0.01) ****
	Biomass/Anthropogenic	1.05 (0.99, 1.11) *	0.004 (0.002) **	1.00 (0.99, 1.01)	−0.04 (0.01) ****
*SUB−REGIONS*					
**East Asia**					
	Biomass	1.02 (1.01, 1.03) **	0.02 (0.004) ****	1.01 (0.99, 1.03)	0.01 (0.01)
	Anthropogenic	1.01 (0.99, 1.04)	0.03 (0.005) ****	0.99 (0.96, 1.02)	−0.002 (0.02)
	Dust	0.98 (0.95, 1.01)	−0.03 (0.006) ****	1.01 (0.97, 1.04)	0.01 (0.02)
	Biomass/Dust	1.00 (0.96, 1.03)	−0.001 (0.001)	0.96 (0.93, 1.01)	−0.05 (0.02) **
	Anthropogenic/Dust	0.96 (0.94, 0.98) ***	−0.02 (0.007) ***	1.01 (0.97, 1.05)	−0.003 (0.02)
	Biomass/Anthropogenic	1.01 (1.00, 1.02) *	−0.02 (0.004) ****	0.99 (0.97, 1.02)	−0.001 (0.01)
**Central/North Asia**					
	Biomass	0.99 (0.99, 1.00)	0.004 (0.005)	1.07 (1.02, 1.13) **	0.07 (0.03) **
	Anthropogenic	1.00 (0.99, 1.01)	−0.01 (0.005) **	1.01 (0.94, 1.08)	−0.02 (0.04)
	Dust	1.01 (1.00, 1.01) **	0.11 (0.008) ****	1.03 (0.97, 1.10)	0.07 (0.03) **
	Biomass/Dust	1.00 (0.99, 1.01)	0.03 (0.007) ****	1.05 (0.96, 1.16)	0.05 (0.05)
	Anthropogenic/Dust	1.00 (0.99, 1.01)	0.003 (0.006)	1.08 (1.00, 1.18) *	0.08 (0.04) *
	Biomass/Anthropogenic	0.99 (0.99, 0.99) ***	−0.04 (0.005) ****	0.96 (0.91, 1.02)	−0.04 (0.03)
**West Asia**					
	Biomass	1.01 (0.99, 1.01)	−0.06 (0.004) ****	0.99 (0.96, 1.03)	−0.01 (0.02)
	Anthropogenic	1.02 (1.01, 1.03) **	0.002 (0.008)	0.89 (0.85, 0.94) ***	−0.14 (0.03) ****
	Dust	0.99 (0.98, 1.00)	−0.10 (0.005) ****	1.03 (0.98, 1.08)	−0.02 (0.02)
	Biomass/Dust	0.96 (0.94, 0.98) ***	0.01 (0.01)	0.82 (0.74, 0.92) ****	−0.13 (0.05) **
	Anthropogenic/Dust	0.99 (0.98, 1.00)	0.03 (0.005) ***	0.94 (0.90, 0.98) ***	−0.01 (0.02)
	Biomass/Anthropogenic	1.01 (1.00, 1.01) **	−0.006 (0.002) **	0.99 (0.96, 1.01)	−0.03 (0.01) **
**South Asia**					
	Biomass	0.90 (0.82, 0.99) *	−0.22 (0.003) ****	0.92 (0.85, 0.99) *	−0.09 (0.02) ****
	Anthropogenic	0.98 (0.89, 1.07)	−0.05 (0.003) ****	1.00 (0.99, 1.00)	−0.005 (0.02)
	Dust	0.97 (0.93, 1.03)	−0.05 (0.002) ****	0.99 (0.95, 1.04)	−0.01 (0.01)
	Biomass/Dust	1.00 (0.95, 1.05)	0.04 (0.002) ****	1.00 (0.99, 1.01)	−0.02 (0.009) **
	Anthropogenic/Dust	0.99 (0.88, 1.12)	−0.10 (0.004) ****	1.00 (0.99, 1.00)	−0.20 (0.02) ****
	Biomass/Anthropogenic	0.95 (0.90, 0.99) *	−0.09 (0.002) ****	0.99 (0.99, 0.99) **	−0.03 (0.009) ****
**Southeast Asia**					
	Biomass	1.07 (0.89, 1.29)	0.02 (0.008) ***	0.96 (0.89, 1.04)	−0.14 (0.03) ****
	Anthropogenic	1.08 (0.94, 1.25)	0.09 (0.006) ****	1.00 (0.99, 1.01)	−0.06 (0.02) **
	Dust	1.05 (0.94, 1.18)	0.03 (0.005) ****	0.98 (0.93, 1.02)	−0.06 (0.02) ***
	Biomass/Dust	1.10 (0.94, 1.28)	0.11 (0.007) ****	1.00 (0.99, 1.01)	−0.02 (0.03)
	Anthropogenic/Dust	1.16 (0.97, 1.38)	0.16 (0.007) ****	1.00 (0.99, 1.01)	0.02 (0.03)
	Biomass/Anthropogenic	1.07 (0.98, 1.16)	0.07 (0.004) ****	0.99 (0.99, 0.99) **	−0.05 (0.01) ****

^a^, In one unit increment of PM_2.5_ concentration. ^b^, Adjusted for country, year, total population, urban population, female population, employed population, HIV/AIDS death, tuberculosis death, undernourished population, and temperature. ^c^, Natural cubit spline smoothing. GLMM + NS, Generalized linear mixed-effect model with natural cubit spline. IRR, Incidence rate ratio. CI, Confidence interval. GAMM, Generalized Additive Mixed Model with natural cubit spline. Coef., Coefficient. *Se*, Standard error. * *p* ≤ 0.10, ** *p* ≤ 0.05, *** *p* ≤ 0.01, **** *p* ≤ 0.001.

## Supplementary Materials

The following are available online at https://www.mdpi.com/1660-4601/16/20/3855/s1, Figure S1: The 2nd-order derivative of spectral aerosol optical depth (AOD) (𝛻^2^τ with 0.47, 0.55 and 0.66 μm related to refractive index) against 1st-order derivative normalized by AOD_(0.47 μm)_ (*NGAI*) simulated with 0.01 step from 0.00 to 1.00 in mixing weights of volume density from 6S model (Second Simulation of a Satellite Signal in the Solar Spectrum, Vermote et al., 2008) [2] for triple-component mixtures (i.e., dust (DS), anthropogenic pollutant (AP) and biomass burning (BB) aerosols). The inputs of optical properties of aerosol models are recommended by the World Meteorological Organization (WMO) [3,4].
