# Supplementary material for: Ambient Particulate Matter Exposure and Under-Five and Maternal Deaths in Asia
Source: Int J Environ Res Public Health. 2019 Oct 12;16(20):3855. doi: 10.3390/ijerph16203855 (PMC6843620; doi:10.3390/ijerph16203855)
Supplement: Supplementary file 1 [file ijerph-16-03855-s001.pdf]

Supplementary Information

The radiometric difference in spectral character between media is principally associated with particle size distribution (1st- order derivative) and imaginary part of the complex refractive index [1]. The spectrum derivative of optical properties can be further linked to the characteristics of suspended particles in scattering and absorption processes (2nd- order derivative). The 1st- and 2nd-order derivatives of spectral aerosol optical depths (AODs), $\mathrm{NGAI}_{(\lambda_1,\lambda_2)}$ and $\nabla^2\tau_{(\lambda_1,\lambda_2,\lambda_3)}$ related to particle size distribution and refractive index of aerosols, can be expressed as equation (1) and (2) as the following,

$$NGAI_{(\lambda_1,\lambda_2)} \equiv \nabla\tau_{(\lambda_1,\lambda_2)} / \tau_{\lambda 1} \quad (1)$$

$$\nabla^2\tau_{(\lambda_1,\lambda_2,\lambda_3)} = \frac{(\tau_{\lambda_1} - 2\tau_{\lambda_2} + \tau_{\lambda_3}) / \tau_{\lambda_1}}{(\lambda_1 - \lambda_2)^2} \quad (2)$$

where $\nabla\tau_{(\lambda_1,\lambda_2)} = \dfrac{\tau_{\lambda_1} - \tau_{\lambda_2}}{\lambda_1 - \lambda_2}$, τ_λ is the AOD at a specific wavelength. The λ_1, λ_2 and λ_3 are the wavelength at 0.47, 0.55 and 0.66 μm, respectively.

In this study, the 1st- and 2nd-order spectral derivatives of aerosol optical depth (AOD), related to particle size and complex refractive index, are explored to characterize their roles using derivative spectroscopy approach (the concept upon spectral mapping) for aerosol partition in detail. With the theoretical simulation from 6S (Second Simulation of a Satellite Signal in the Solar Spectrum) model, AOD Spectral derivatives are characterized for each aerosol type and stretched out for partitioning aerosols between mineral dust (DS), biomass burning (BB) particle, anthropogenic pollutant (AP) and the mixtures of them, see also Figure S1. The major component(s) of pure (mixed) aerosols thus can be identified (partitioned).

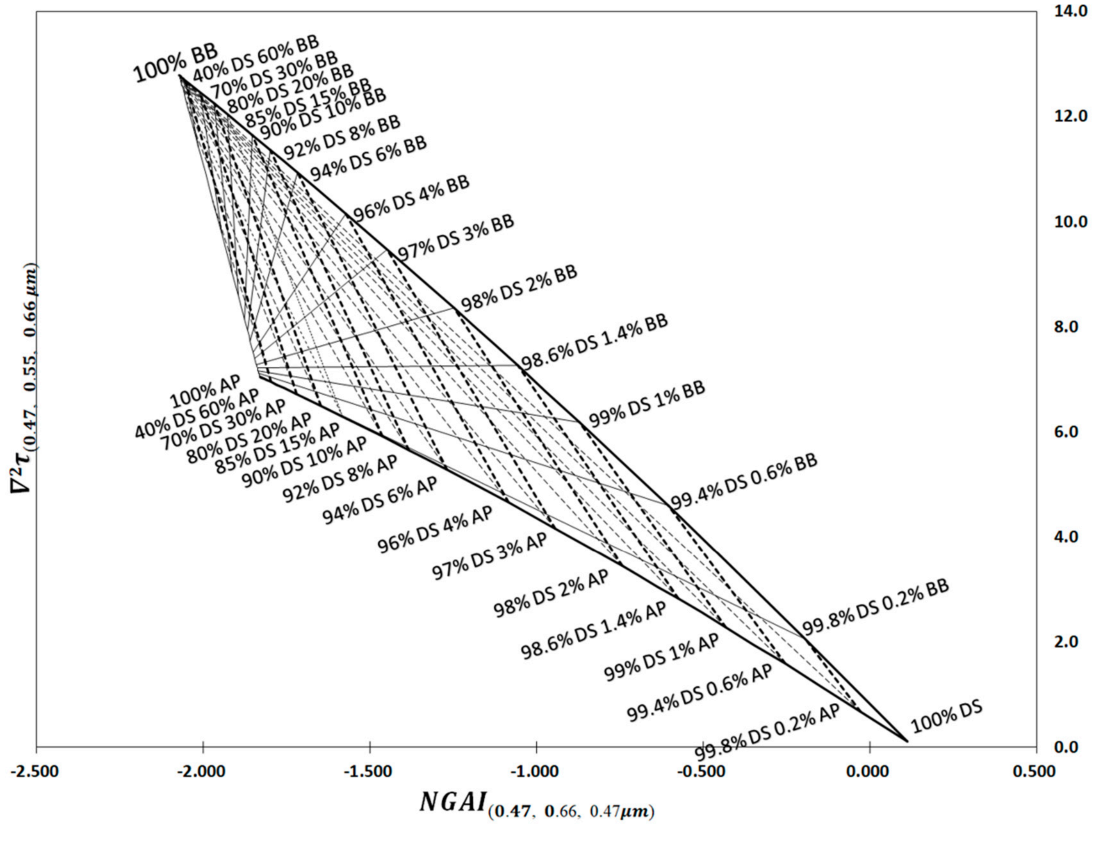

Figure S1. The 2nd-order derivative of spectral aerosol optical depth (AOD) ($\nabla^2\tau$ with 0.47, 0.55 and 0.66 μm related to refractive index) against 1st-order derivative normalized by $AOD_{(0.47\ \mu m)}$ (*NGAI*) simulated with 0.01 step from 0.00 to 1.00 in mixing weights of volume density from 6S model (Second Simulation of a Satellite Signal in the Solar Spectrum, Vermote et al., 2008) [2] for triple-component mixtures (i.e., dust (DS), anthropogenic pollutant (AP) and biomass burning (BB) aerosols). The inputs of optical properties of aerosol models are recommended by the World Meteorological Organization (WMO) [3,4].

References

1. Hansell, R. A.; Tsay, S.-C.; Pantina, P.; Lewis, J. R.; Ji, Q.; Herman J. R. Spectral derivative analysis of solar spectroradiometric measurements: Theoretical basis. *J. Geophys. Res. Atmos.* **2014,** *119,* 8908–8924, doi:10.1002/2013JD021423.
2. Vermote, E. F.; Kotchenova, S. (2008). Atmospheric correction for the monitoring of land surfaces. *J. Geophys. Res: Atmos.* **2008,** *113,* D23S90, doi:10.1029/2007JD009662.
3. Deepak, A.; Gerber, H. E. *Report of the experts meeting on aerosols and their climatic effects, World Climate Programme,* ***55***. World Meteorological Organization: Geneva, Switzerland. 1983.
4. McClatchey, R. A.; Bolle, H. J.; Kondratyev, K. Y.; Joseph, J. H.; McCormick, M. P.; Raschke, E.; Pollack, J. B.; Spankuch, D.; Mateer, C.; Shettle, E., et al. *A preliminary cloudless standard atmosphere for radiation computation.* World Meteorological Organization: Colorado, USA, 1984.
